# Tuberculosis screening for pediatric household contacts in India: Time to adapt newer strategies under the National TB Elimination Programme!

**DOI:** 10.1371/journal.pone.0292387

**Published:** 2023-10-05

**Authors:** Kiran Chawla, Sharath Burugina Nagaraja, Nayana Siddalingaiah, Chidananda Sanju, Uday Kumar, Vishnu Prasad Shenoy, Suresh Shastri, Anil Singarajipur

**Affiliations:** 1 Department of Microbiology, Kasturba Medical College Manipal, Manipal Academy of Higher Education Manipal, Manipal, Karnataka, India; 2 Employees State Insurance Corporation Medical College and Post Graduate Institute of Medical Sciences and Research, Bengaluru, Karnataka, India; 3 District Tuberculosis Office, Udupi, Karnataka, India; 4 State Tuberculosis Office, Bengaluru, Karnataka, India; Faculty of Life and Allied Health Sciences, Ramaiah University of Applied Sciences, INDIA

## Abstract

**Introduction:**

The study aimed to evaluate the effectiveness of screening pediatric household contacts (under the age of 15 years) for tuberculosis (TB) in India through verbal screening, tuberculin skin testing, and chest radiography at intervals of 0, 3, 6, 9, and 12 months. The study also aimed to determine the proportion of contacts who tested positive for TB and to describe the challenges in implementing regular follow-up. Current National TB Elimination Programme (NTEP) guidelines only require verbal screening for contacts under 6 years old at TB treatment initiation. The study aimed to fill this knowledge gap and provide valuable insights for improving TB screening in pediatric household contacts in India.

**Methods:**

The study was conducted in two districts of Karnataka, India from 2021 to 2022, and utilized a cohort study design to enroll contacts of index tuberculosis (TB) cases diagnosed under the National TB Elimination Programme (NTEP). Participants were followed up at regular intervals for one year to evaluate the effectiveness of TB screening in pediatric household contacts.

**Results:**

In this study, 686 pediatric household contacts were enrolled and screened for tuberculosis (TB) using verbal symptom screening, tuberculin skin testing (TST), and chest radiography. Projected figures estimated that 0.8%, 42%, and 4% of contacts would test positive for symptomatic screening, TST, and chest radiography, respectively. TB cases were detected in 2.91% (1.84–4.38) of contacts, with females above 6 years of age having a 22% higher risk of contracting the infection than males above 6 to < 15 years. However, not all cases were subjected to TST and chest radiography. The primary reason for not investigating child contact for TB was their reported healthy or asymptomatic status.

**Conclusion:**

The implementation of regular screening intervals for tuberculin skin test (TST) and chest radiography, along with verbal screening, among pediatric household contacts under the age of 15 years seems to be beneficial for the National TB Elimination Programme (NTEP), despite the challenges faced during implementation. Innovative strategies should be explored by NTEP to ensure effective implementation.

## Introduction

In 2015, the World Health Organization (WHO) launched ‘The End TB strategy’ to eliminate Tuberculosis (TB) globally, with a key goal of treating 3.5 million children with TB disease [1]. However, only 54% of children with TB disease were treated from 2018 to 2022. The COVID-19 pandemic caused a steep decline in TB notifications globally, with India accounting for nearly 67% reduction in total TB case notifications in 2020 [1, 2]. Childhood TB has been a staggering public health concern, especially in high-burden countries. Globally 33% of childhood TB cases are notified from India despite of gap between the estimated TB cases in children to notified cases being about 56% giving an idea of the number of cases that are undiagnosed [3]. Of all the TB cases notified under the National TB Elimination Program (NTEP) in India, nearly 7% are pediatric TB cases [1]. NTEP has taken measures to control and prevent the spread of TB, including screening and implementing tuberculosis preventive treatment (TPT) for household contacts of bacteriologically confirmed TB cases aged less than six years. However, according to the WHO Global TB Report (2022), only 40% of pediatric household contacts aged below five years are initiated on the TPT [1]. Studies show that tuberculosis prevalence is higher in children with a positive Tuberculin Skin Test (TST) or Interferon Gamma Release Assay (IGRA) than those with negative results. It is important to ensure that pediatric household contacts are not missed, as they are at a higher risk of developing TB infection or disease. Screening with other tools like TST, chest radiography or cartridge-based nucleic acid amplification test (CB-NAAT) at regular intervals of three months can be useful in identifying and monitoring these contacts for better treatment outcomes. This study aimed to determine the proportion of pediatric household contacts who are likely to be positive for TB infection or disease on screening using different tools at regular intervals of three months under programmatic settings and to describe the challenges encountered during project implementation.

## Methods

The study employed a cohort design to investigate the burden of tuberculosis (TB) among pediatric household contacts of NTEP-notified TB patients who initiated TB treatment in the Bengaluru and Udupi districts of Karnataka, India, between January 2021 and June 2022. The study population was followed up for a period of one year, until December 2022 (Follow-up duration varied based on enrolment, children enrolled from January 2022-June 2022 were followed up till the study concluded). Verbal symptom screening, chest radiography, and tuberculin skin testing (TST) were used to detect TB infection or disease among pediatric household contacts aged less than 15 years, at baseline, 3rd, 6th, 9th, and 12th month of follow-up. The third and ninth-month follow-up encompassed telephonic discussions with the child’s guardian or parent to conduct symptom screening. Conversely, during the sixth and twelfth-month follow-ups, the child physically visited the medical facility for diagnostic assessments and underwent symptom screening conducted by a qualified paediatrician. Trained project staff were recruited for the study to facilitate the follow-up process.

### The National TB Elimination Program in Karnataka

Karnataka is a state located in southern India with a population of approximately 67.2 million [4]. It is divided into 31 administrative districts, and in 2020, it notified 3.3% of total TB cases in India. From July 2021 to June 2022, a total of 79,537 TB cases were reported in Karnataka, with the Bengaluru district contributing to 19% (15,222/79,537) of cases, and the Udupi district contributing 1.8% (1,455/79,537). Bengaluru is the capital city of Karnataka, with a population of over 11.6 million, and it has 32 Tuberculosis Units (TU), which are the programmatic management units under the National Tuberculosis Elimination Program (NTEP) covering a population of approximately 0.25 million. For this study, 14 TUs were selected in the Bengaluru district using a convenient sampling method. The Udupi district, which has a population of 1.2 million and six TUs, was included in the study, and all TUs were selected. Both districts have a robust public and private health sector, but the public sector predominates in delivering TB services in the community.

### Inclusion criteria for pediatric household contacts

The study included all pediatric household contacts who were under 15 years of age and resided within the study area, and who had been in contact with confirmed TB patients, with or without a history of TB preventive therapy. Pediatric household contacts who were receiving anti-TB treatment and parents or guardians who did not provide written informed consent were excluded from the study. The process for verbal symptom screening, TST and chest radiography is elaborated below:

(a) *Verbal symptom screening* for four TB symptom complex: The paediatric household contacts were screened for following symptoms:

1. Cough: A persistent cough that lasts for more than two weeks is one of the most common symptoms of TB. The cough may be dry or productive and may be accompanied by phlegm or blood.
2. Fever: A low-grade fever that persists for several weeks may be a sign of TB. The fever may be accompanied by night sweats and chills.
3. Weight loss: Unexplained weight loss of more than 3–4 kilograms over a few weeks or months can be a sign of TB.
4. Night sweats/fatigue: Night sweats and chronic fatigue or weakness are also common symptoms of TB. People with TB may feel tired and run down even after getting enough rest.

(b) *Tuberculosis Skin test (TST)*

For the study, tuberculin skin testing (TST) was conducted at government hospitals or private diagnostic centers following standard protocols [5]. Project staff members made all necessary arrangements to ensure that household contacts could be mobilized to these centers, and that patients did not incur any costs for the tests. Protein Purified Derivative (PPD) strength for the test varied between centers, and included 1 TU, 2 TU, 5 TU, and 10 TU depending on availability. Trained laboratory technicians measured the induration after 48–72 hours, and results were subsequently validated by senior pediatricians at the study sites. A positive test result was defined as any induration greater than 10 millimeters [6].

(c) *Chest radiography*

Chest radiography was conducted at government hospitals or private diagnostic centers that were identified for the study. Radiology reports were provided by radiologists and further evaluated by pediatricians. Diagnosis by the pediatrician was based on the clinical profile of the contacts as reported by project staff, as well as the radiography findings. In cases where the disease was highly suspected, all possible efforts were made to bring pediatric contacts to the pediatrician. If required, further evaluations were conducted at the hospital on the advice of the pediatrician. For those cases, samples were collected using induced sputum or bronchoalveolar lavage and subjected to CB-NAAT analysis.

### Follow-up at regular interval

Following baseline screening, pediatric household contacts were regularly screened at 6-month intervals for up to one year using verbal symptom screening, tuberculin skin test (TST), and chest radiography. TST was performed only at baseline and at 6 months. The study sought the assistance of grassroots-level health workers from the general health system to ensure regular follow-up of pediatric household contacts. Project staff and program staff actively reminded and motivated caretakers to take the household contacts for screening at designated testing centers, based on their convenience. All costs incurred for investigations and transport were reimbursed to patients by the project.

### Sample size

The sample size was determined based on the assumption that 2% of pediatric tuberculosis (TB) household contacts would develop TB disease within one year. The sample size calculation was performed using www.openepi.com (which is a freely available online software) with a relative precision of 1%, a power of 80%, a confidence interval of 95%, and a design effect of 1 [7]. The estimated sample size for the study was 753 participants.

### Sampling technique

The study included all household contacts under the age of 15 years who were in contact with newly diagnosed, confirmed tuberculosis (TB) patients, regardless of their symptoms or previous history of preventive therapy for TB, and who resided within the two districts under investigation.

### Data collection

The data was collected from participants using a pre-tested and pre-designed proforma. Trained field staff performed data collection during symptom screening, baseline investigation, and follow-up visits. A senior trained researcher in the field validated approximately 10% of the available data to ensure accuracy. The data was then entered into a Microsoft Excel sheet and curated to obtain the final clean dataset for analysis.

### Data analysis

The data was analysed for descriptive statistics like frequency, proportion, percentiles, confidence intervals and relative risk using the statistical software STATA 17.0 BE-Basic (Serial number: 301706309069). The missing data were excluded from analysis. We conducted a sensitivity analysis to assess the potential impact of varying the assumptions obtained from the study results on the projected figures.

### Ethics

The study was submitted to and approved by two institutional ethics committees: ESIC Medical College and PGIMSR (approval no. 523/L//11/12/Ethics/ESIC Medical College and PGIMSR, Bengaluru, India) and Kasturba Medical College, Manipal Academy of Higher Education (approval no. 626/2022).

## Results

Out of the total 1424 eligible pediatric household contacts residing with the index TB cases, 686 individuals, which accounts for 48%, consented to participate in the study.

### Clinico-demographic characteristics of paediatric household contacts

**Fig 1:**
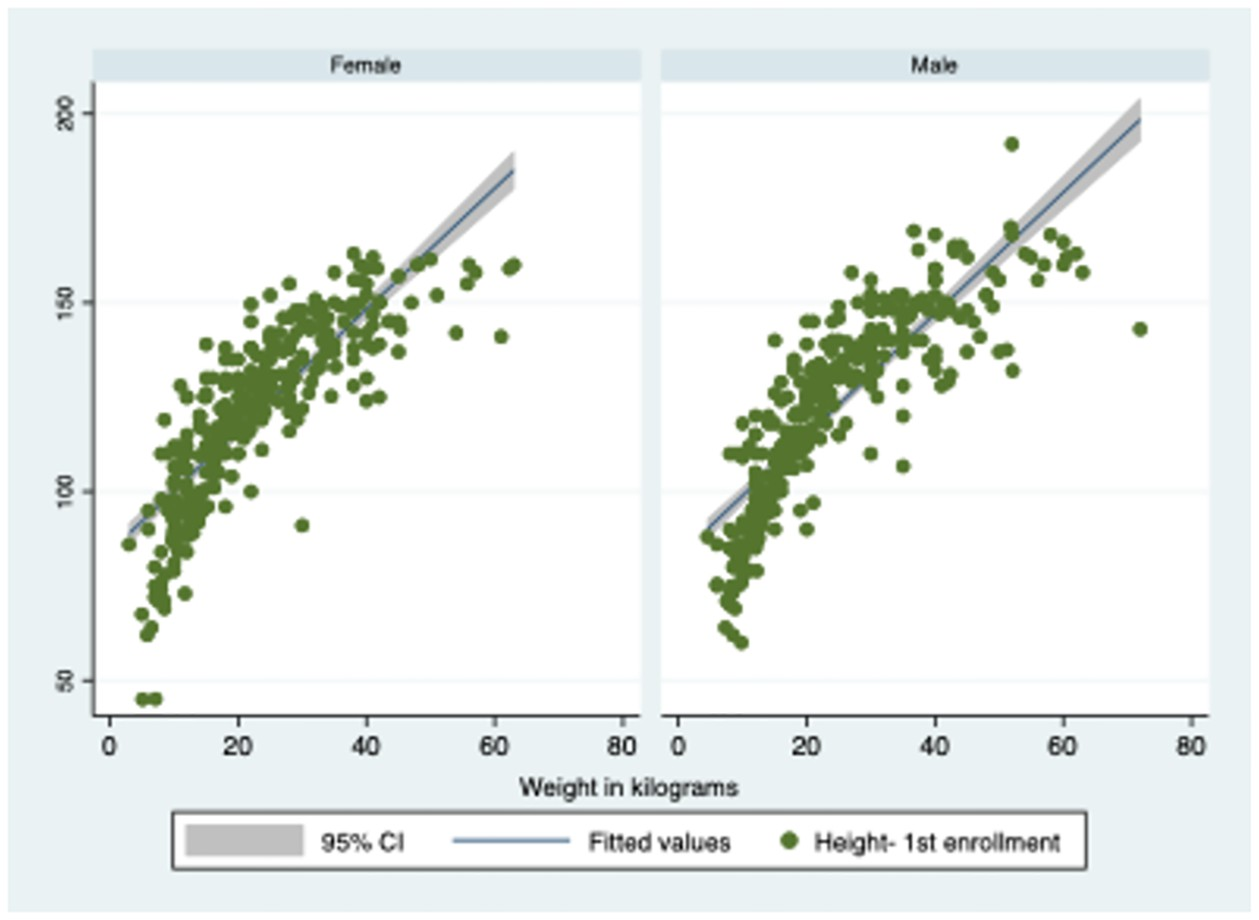
Distribution of study population stratified by gender.

A total of 367 index tuberculosis (TB) cases were enlisted for investigation in two districts. The study identified 686 household contacts under the age of 15 years who were exposed to the index cases, with an average age of 7.61 years and a standard deviation of 4.06. The study successfully enrolled 91% (686/753) of the targeted sample size. Among the identified contacts, 245 (36%) were under the age of 6 years, while 441 (64%) were over 6 years old. The distribution of paediatric household contacts for height, weight, and TST (tuberculin skin test) was similar in both age groups, as shown in Table 1 and Fig 1.

**Table 1:** Percentiles and centiles for height, weight and TST results stratified by age less than and more than 6 years.

		**Less than 6 years(n = 222)**		**More than 6 to < 15 years (n = 441)**	
		Male(n = 112)	Female (n = 110)	Male(n = 200)	Female(n = 211)
**Height**	Percentile	Centile (95% CI)	Centile (95% CI)	Centile (95% CI)	Centile (95% CI)
	25	85.25(80–90)	88.75(84–91)	125(121–128)	123(121–125)
	50	96.50 (92–100)	97.25(95–101)	137(133–140)	134(130–137)
	75	109(104–112)	110(106–112)	148(145–150)	145(142–147)
		Less than 6 years(n = 229)		More than 6 to < 15 years (n = 421)	
		Male(n = 115)	Female (n = 114)	Male(n = 206)	Female(n = 215)
**Weight**	Percentile	Centile (95% CI)	Centile (95% CI)	Centile (95% CI)	Centile (95% CI)
	25	10 (9–10.6)	10 (8–10)	21(20–23)	21(20–22)
	50	12 (12–13)	12 (11–13)	29 (26–30)	27 (26–29)
	75	15 (14–16)	15 (14–16)	38 (35–40)	35 (33–38)
		Less than 6 years(n = 54)		More than 6 to < 15 years (n = 129)	
**TST**	Percentile	Centile (95% CI)		Centile (95% CI)	
	25	13(12–14.5)		14(13–15)	
	50	16 (14.2–18)		18 (16–18)	
	75	20 (18–21)		20 (20–22)	

#### Household contacts less than 6 years

Among the paediatric household contacts, roughly 50% of males were below 96.50 centimetres in height, while approximately 50% of females were below 97.25 centimetres. Additionally, about 50% of both male and female paediatric household contacts had a weight below 12 kilograms.

#### Household contacts more than 6 to < 15 years

Around 50% of the male paediatric household contacts had a height below 137 centimetres, whereas roughly 50% of the female contacts had a height below 134 centimetres. In addition, approximately half of the male and female paediatric household contacts had a weight below 29 kilograms and 27 kilograms, respectively.

**Fig 2:**
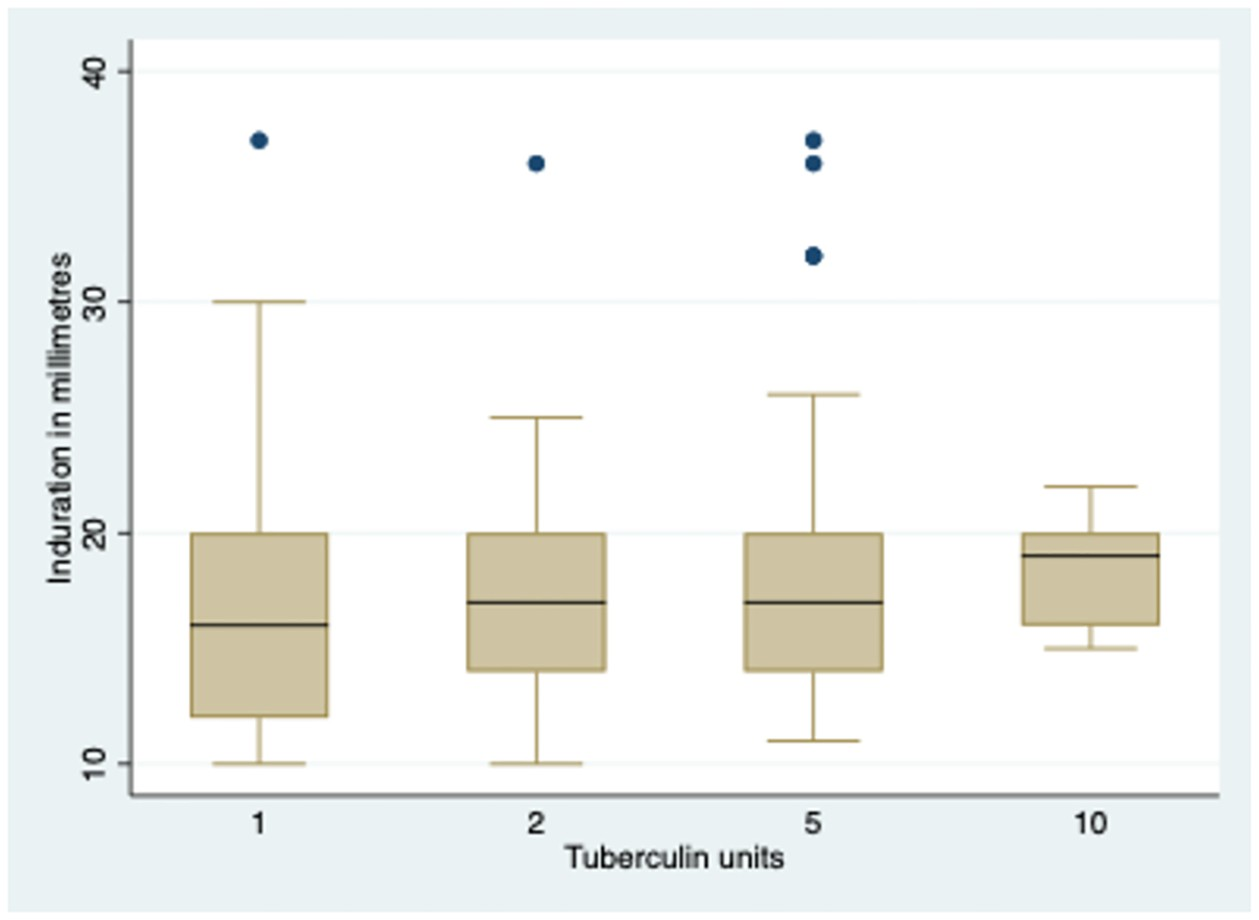
Box Plot showing the TST results for different Tuberculin units.

**Fig 3:**
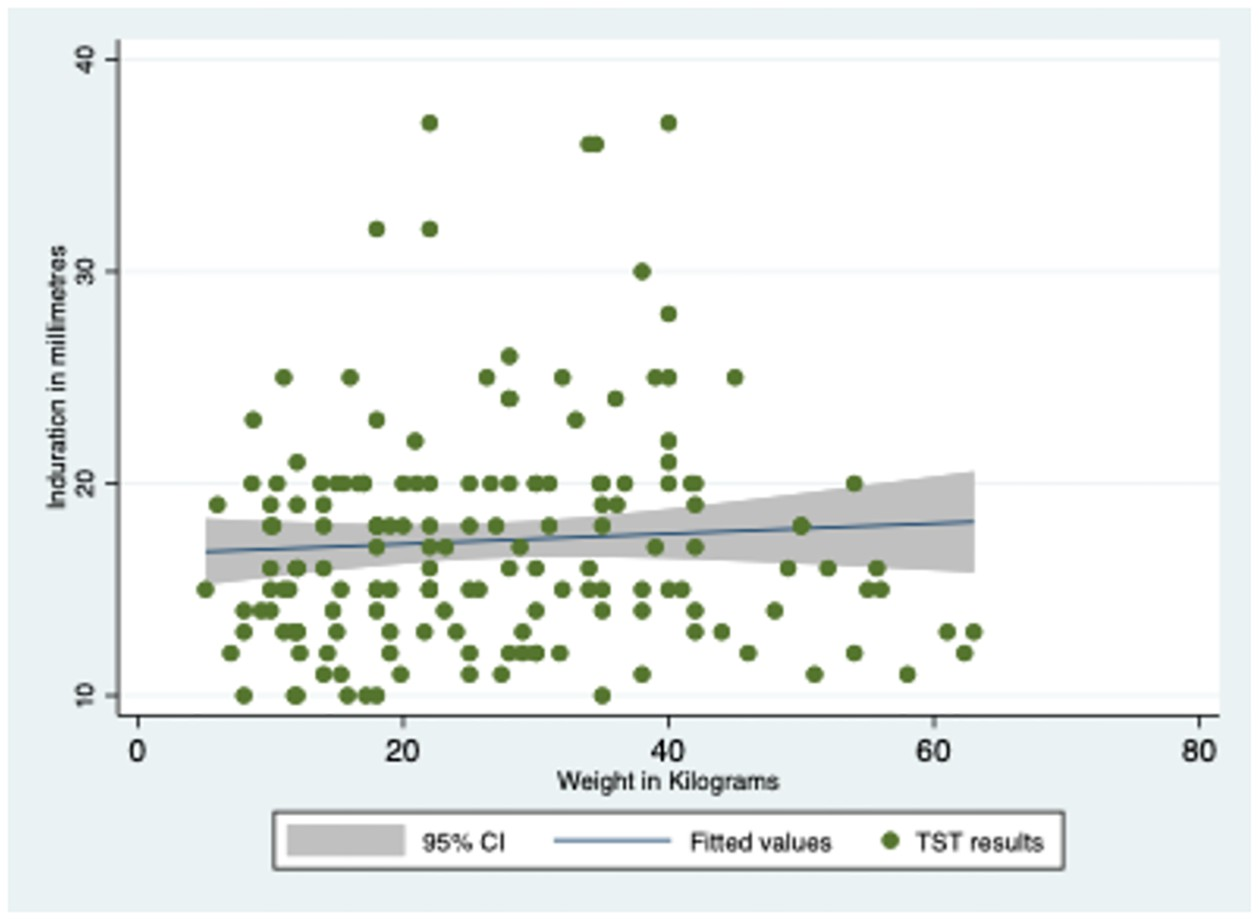
TST results and weight of the study population.

**Fig 4:**
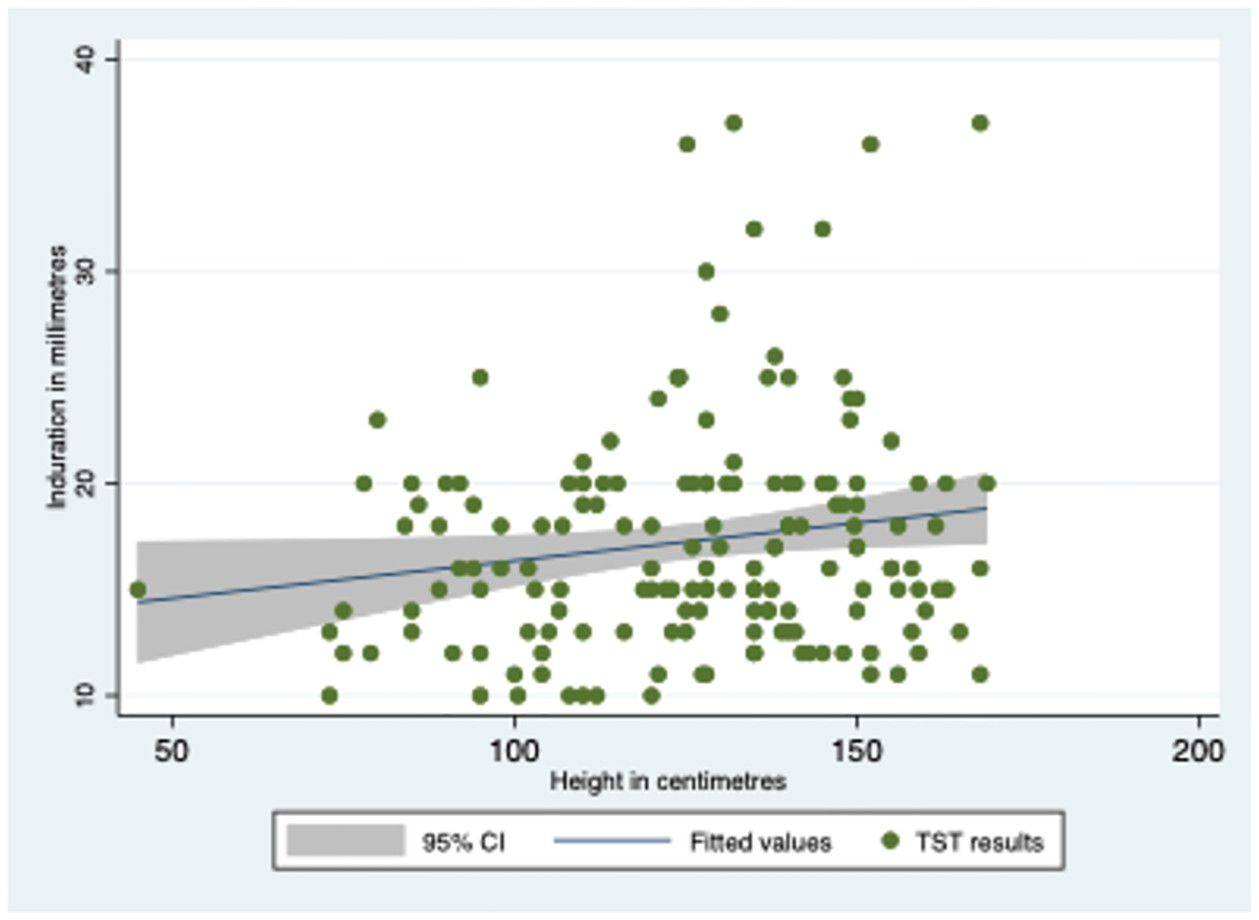
TST results and height of the study population.

**Fig 5:**
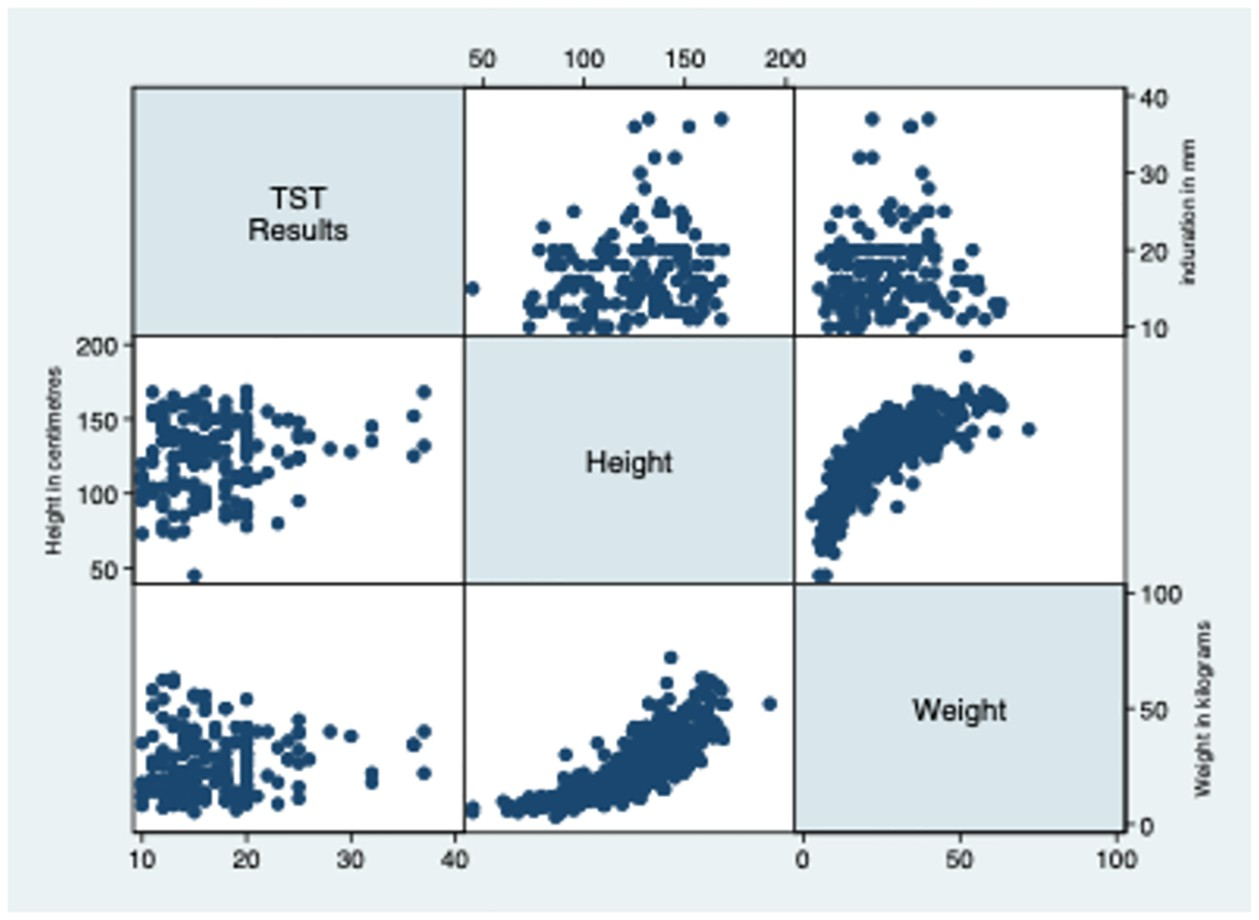
Graph Matrix showing the distribution of TST results, height, and weight of the study population.

The average induration size of individuals with a TST result above 10 mm was 17.66±5.30 mm, as revealed in Table 2 and Fig 2. Most of the participants were subjected to a 5 TU test, and the mean induration size was 17.80 ± 5.12 mm. Notably, no correlation was observed between TST results and the weight or height of the study population, as shown in Figs 3 and 4. The TST results, weight, and height graph matrix is depicted in Fig 5.

**Table 2:** Tuberculosis Skin Test (TST) results stratified by strengths (1,2,5 and 10) of Tuberculosis Units (N = 183).

Tuberculosis unit	Observations	Mean *(millimetre)*	Standard deviation	Minimum	Maximum
1	37	17.16	6.15	10	37
2	22	17.40	5.86	10	36
5	114	17.80	5.12	11	37
10	10	18.40	2.40	15	22
Overall	183	17.66	5.30	10	37

#### Follow-up of paediatric household contacts on symptom screening, chest radiography and TST

Within the group of contacts, males and females comprised approximately 49% and 51%, respectively, totalling 338 and 348 individuals. Using these proportions, we have extrapolated the positivity rates to estimate the number for the entire cohort. Tables 1–3 provide information on the frequency of testing, as well as the number of paediatric household contacts screened and identified as positive for verbal symptom screening, TST, and chest radiography, respectively.

**Table 3:** Verbal symptom screening at regular intervals and their positive results (N = 686).

Frequency of testing	Symptom screening n(%)	Result Positives n(%)	Projected figures n(%)
Baseline	686 (100)	21/686 (3)	21(3)
3^rd^ month	659 (96)	0/659 (0)	0(0)
6^th^ month	616(90)	0/616 (0)	0(0)
9^th^ month	475 (70)	0/475 (0)	0(0)
12^th^ month	415(61)	1/415 (0.2)	2(0.2)
Total	2851	22(0.7)	23(0.8)

Based on the estimated figures, the results indicate that roughly 0.8% of paediatric household contacts were identified as positive for verbal screening over the entire project duration, as indicated in Table 3.

The projected data indicates that approximately 42% of paediatric household contacts were identified as positive for TST throughout the entire project duration, as presented in Table 4.

**Table 4:** TST screening at regular intervals and their positive results (N = 686).

Frequency of testing	TST screening n(%)	Result Positives n(%)	Projected Figures n(%)
Baseline	686 (100)	183/686 (27)	183(27)
6^th^ month	182 (27)	49/182 (27)	183(27)
12^th^ month	10 (2)	0/10 (0)	0(0)
Total	878	232/878 (26.4)	366(42)

Based on the estimated figures, the results indicate that almost 4% of paediatric household contacts displayed abnormal chest radiography throughout the project duration, as shown in Table 5.

**Table 5:** Chest radiography screening at regular intervals and their positive results (N = 686).

Frequency of testing	Chest radiography screening n(%)	Abnormal results n(%)	Projected Figures n(%)
Baseline	671 (98)	20/671 (3)	21(3)
6^th^ month	182 (27)	04/182 (2)	14(2)
12^th^ month	10 (2)	0/10 (0)	0(0)
Total	863	24/863(2.7)	35(4)

In total, our study revealed that 424 out of 686 contacts (62%) tested positive for at least one of the screening tests over the course of the study. It is important to note, however, that these figures include household contacts who underwent one or multiple screenings. Furthermore, less than 0.1% of contacts completed all the screening tests as scheduled and received all of them.

#### Clinico-demographic characteristics of paediatric household contacts found to be positive for TB

In total, 20 cases of TB were identified through screening out of 686 individuals, resulting in a proportion of 2.91% (1.84–4.38). Among these cases, 80% (16 out of 20) were individuals over the age of 6 to < 15 years. Of those over six years old, 41% (5 out of 12) were tested positive for TST. Additionally, females above six years old were found to be at 22% risk of contracting the infection when compared to males above 6 to < 15 years of age. Some contacts were not examined for TST, radiography, or CB-NAAT due to their unwillingness or lack of advice, as presented in Table 6.

**Table 6:** Clinicodemographic characteristics of the paediatric contacts found to be TB-positive on screening.

Characteristics	< 6 years [n = 04] (%)	6 to <15 years [n = 16] (%)	Relative Risk
**Gender**			1.22
**Male**	02 (50)	07(44)	
**Female**	02(50)	09(56)	
**Previous IPT administration**	01/04(25)	00	
**Symptom positive**	00	04(25)	
**TST positive**	04/04 (100)	05/12* (41)	
**Mean TST diameter (millimetre)**	18	18	
**Abnormal chest radiography**	01/04 (25)	07/15* (46)	

* Few children did not undergo TST and chest radiography

#### Reasons for non-compliance to follow-up

Table 7 displays the reasons provided by parents of paediatric household contacts for not having their child undergo TST or chest radiography. Among those who did not have their child undergo screening, the majority (46%) believed that their child did not exhibit any symptoms, while 12% were unwilling to perform the tests.

**Table 7:** The reasons for not getting the child investigated for TST or Chest radiography as opined by parents or guardians [N = 504].

Reasons	N (%)
The child had no symptoms of TB	231 (46)
Parents of children were not willing to the investigation	60 (12)
Children were shifted to a different household or place	50 (10)
The guardian or parent was unable to connect on a call	42 (8)
Children had schools or exams or tuitions	39 (8)
Parents of children didn’t respond to calls	31 (6)
Working parents didn’t have a holiday	20 (4)
Visited the hospital but the investigation was not carried out as the child is asymptomatic	20 (4)
Children who were already on ATT treatment	11 (2)

## Discussion

This study, conducted in India, is unique in that it aimed to screen and evaluate a cohort of paediatric household contacts (under the age of 15) through symptom screening, tuberculin skin tests, and chest radiography for a duration of one year. The findings indicate that nearly 50% of paediatric household contacts tested positive for TB infection, and of the 686 individuals screened, 3% (20) were diagnosed with TB. Furthermore, 80% of these TB cases were observed in the 6 to < 15 years age group.

The programmatic implications of our study findings are described under the below headings.

### Clinico-demographic characteristics of paediatric household contacts

In comparison to the general population, the height and weight of the paediatric household contacts did not differ significantly [8]. Among those below the age of 6 years, there was no difference in the height and weight of males and females. However, for those above the age of 6 to < 15 years, the males showed a slightly better height and weight, possibly due to the pubertal growth spurt in males. Therefore, the commonly held belief that paediatric household contacts of tuberculosis patients are malnourished or undernourished may not be accurate.

Among the TST-positive cases, nearly 50% had mean induration between 16–18 mm, which is higher than the standard cut-off of 10 mm in India [9]. This finding needs further investigation as it deviates by 6–8 mm from the usual cut-off. Additionally, the strength of TU used for TST can affect the induration, and the majority of TSTs in the district were done using 5 TU. The higher mean cut-off for higher tuberculin strengths also needs to be explored. The availability of similar strengths of tuberculin across the district needs to be made uniform as a policy. Our study found that TSTs were mainly performed at private laboratories by laboratory technicians, and the quality of TSTs done at private sector is unmonitored. The high technical skills required for the test and lack of monitoring could lead to varying results among the contacts. Furthermore, the storage conditions of the purified protein derivative used to perform these tests are also not monitored. These factors could significantly affect patient management in both public and private sectors.

In screening for tuberculosis, access to chest radiography is crucial. Unfortunately, not all public health facilities have the necessary equipment for radiography, and even if they do, priority may be given to emergency cases over asymptomatic children. Functional radiography is often only available at private facilities, and parents may be reluctant to spend money on screening their asymptomatic child. Additionally, there may be limited capacity to interpret chest radiography results at health facilities. To address these issues, there is a pressing need for the program to improve access to health facilities and develop a training module on "Radiological findings in Tuberculosis."

### Follow-up of paediatric household contacts on symptom screening, chest radiography and TST

The COVID-19 pandemic has made follow-up testing of paediatric household contacts challenging, particularly in terms of symptom screening, chest radiography, and tuberculin skin tests (TSTs). Apart from this many parents opined that their child had no symptoms and many of them were hesitant to subject their children to unnecessary investigations. To estimate the potential positivity rates for these tests, the study projected figures based on its sample size and results, as it was not feasible to perform all three tests three times a year as per the study protocol. The projected figures indicated that around 0.8%, 42%, and 4% of contacts would test positive for symptomatic screening, TST, and chest radiography, respectively. Notably, the TST positivity rate was lower than that found in Malawi [6]. Currently, the National Tuberculosis Elimination Programme (NTEP) in India screens only children under six years of age once, during TB patient registration for treatment. We suggest that the screening policy should be revised to include at least three screenings a year with symptom screening, chest radiography, and TST or any other non-invasive test for latent TB infection. We would like to emphasize the importance of multiple screenings, as the disease may not always present with symptoms, TST positivity, or abnormal chest radiography. Hence, we recommend that contacts who test positive on initial screening be evaluated for TB disease and managed appropriately and urge NTEP to adopt a new screening policy for household contacts to ensure that no TB infection or case goes undetected under the programme.

### Reasons for non-compliance to follow-up

Overcoming the challenge of subjecting an asymptomatic child to various tests to rule out TB infection and disease is crucial. Innovative strategies must be adopted to raise awareness among the community and the parents of index TB cases.

## Strengths and limitations

The strength of the study is that it was carried out in programmatic conditions, accurately reflecting real-world conditions, and it also adhered to the STROBE guidelines [10]. The study had several limitations. Firstly, due to the COVID-19 pandemic and hesitancy by the parents, not all contacts were able to undergo all three screening tests at 0, 6, and 12 months, which limited the completeness of the data. Hence, the findings have to be interpreted with great caution. Secondly, the strength of tuberculin used for TST varied, making it difficult to draw general conclusions about the results. Thirdly, the size of induration was only recorded for positive TST results (greater than 10 mm), while negative results (less than 10 mm) were not measured and were reported as negative, which prevented the calculation of a mean induration for all TST results. Fourthly, the findings of chest radiography were not quantified beyond normal or abnormal categories. Finally, there was no policy in place to screen contacts at regular intervals, and some patients were hesitant to undergo testing.

## Conclusion

In the current era of tuberculosis elimination, screening of paediatric household contacts under the age of 15 years using symptom screening, TST and chest radiography at 0, 6th and 12th month seems to be important for early diagnosis of paediatric tuberculosis cases. However, further research in this direction is needed as our study could not follow-up all cases in totality as envisaged. Uniformity is usage of TST strength and approachability of radiology services in NTEP program is highly recommended. It is important to note that even a single case of TB is significant and hence, the National Tuberculosis Elimination Program (NTEP) should prioritize the development of new policies and strategies for the screening of paediatric contacts.

## Supporting information

S1 Data set (XLSX)
